# Supplementary figures and images for: Effect of Biogas Slurry on the Soil Properties and Microbial Composition in an Annual Ryegrass-Silage Maize Rotation System over a Five-Year Period
Source: Microorganisms. 2024 Apr 1;12(4):716. doi: 10.3390/microorganisms12040716 (PMC11051864; doi:10.3390/microorganisms12040716)

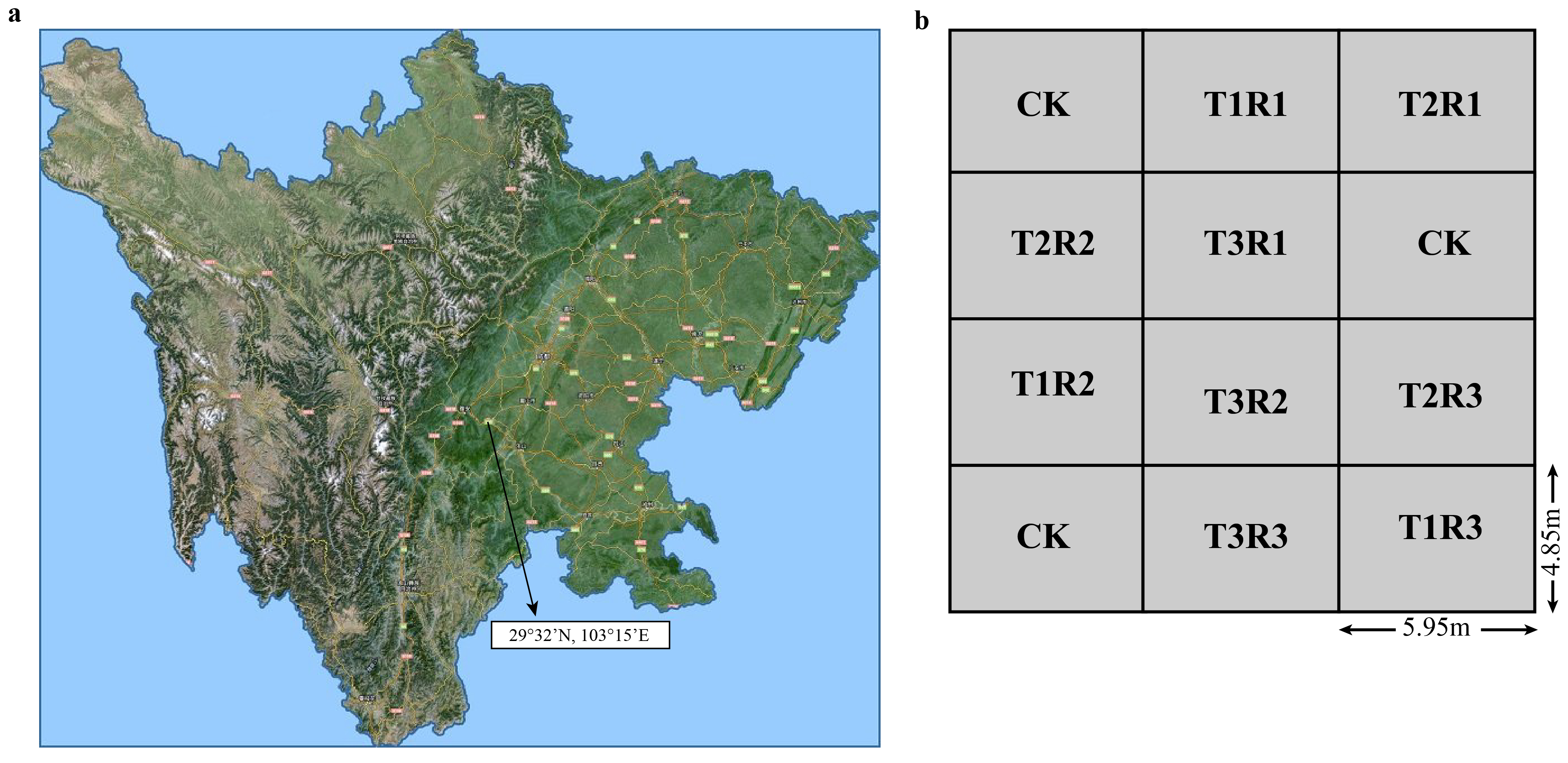

Supplement: Supplementary file 1 [file microorganisms-12-00716-s001.zip › manuscript-supplementary/Figure S1.jpg]

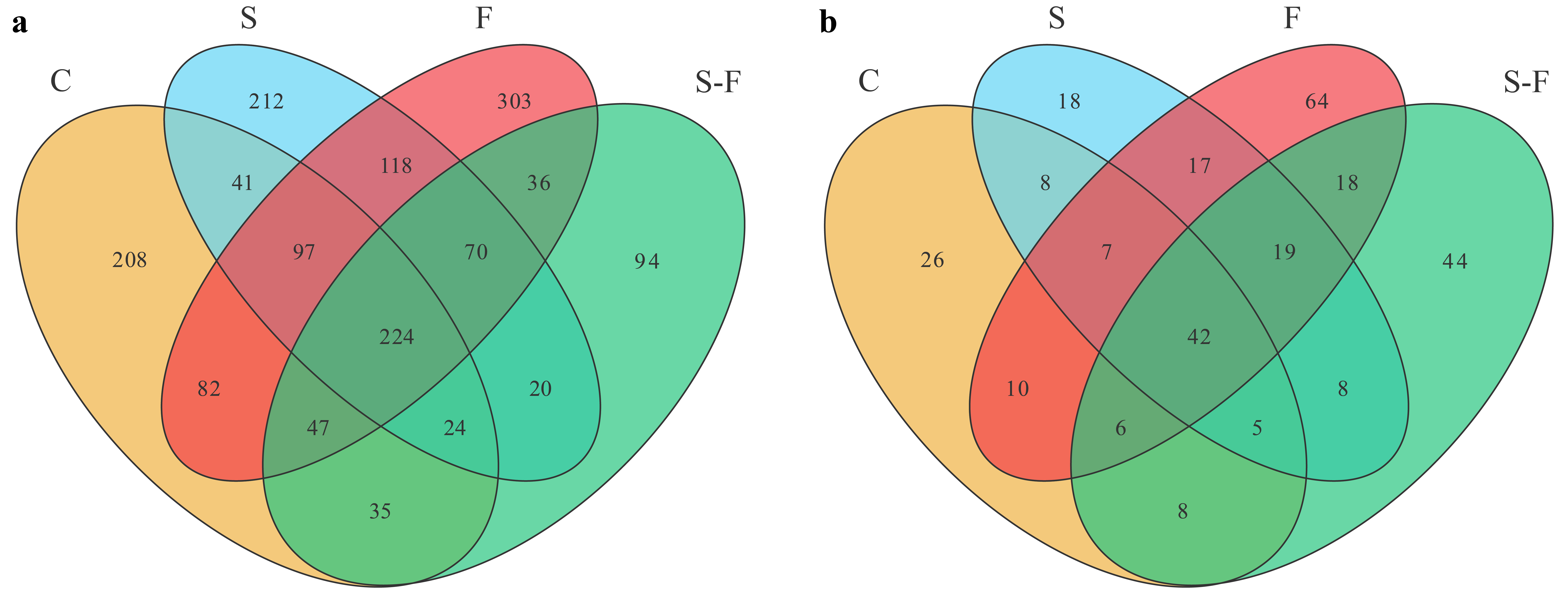

Supplement: Supplementary file 1 [file microorganisms-12-00716-s001.zip › manuscript-supplementary/Figure S2.jpg]

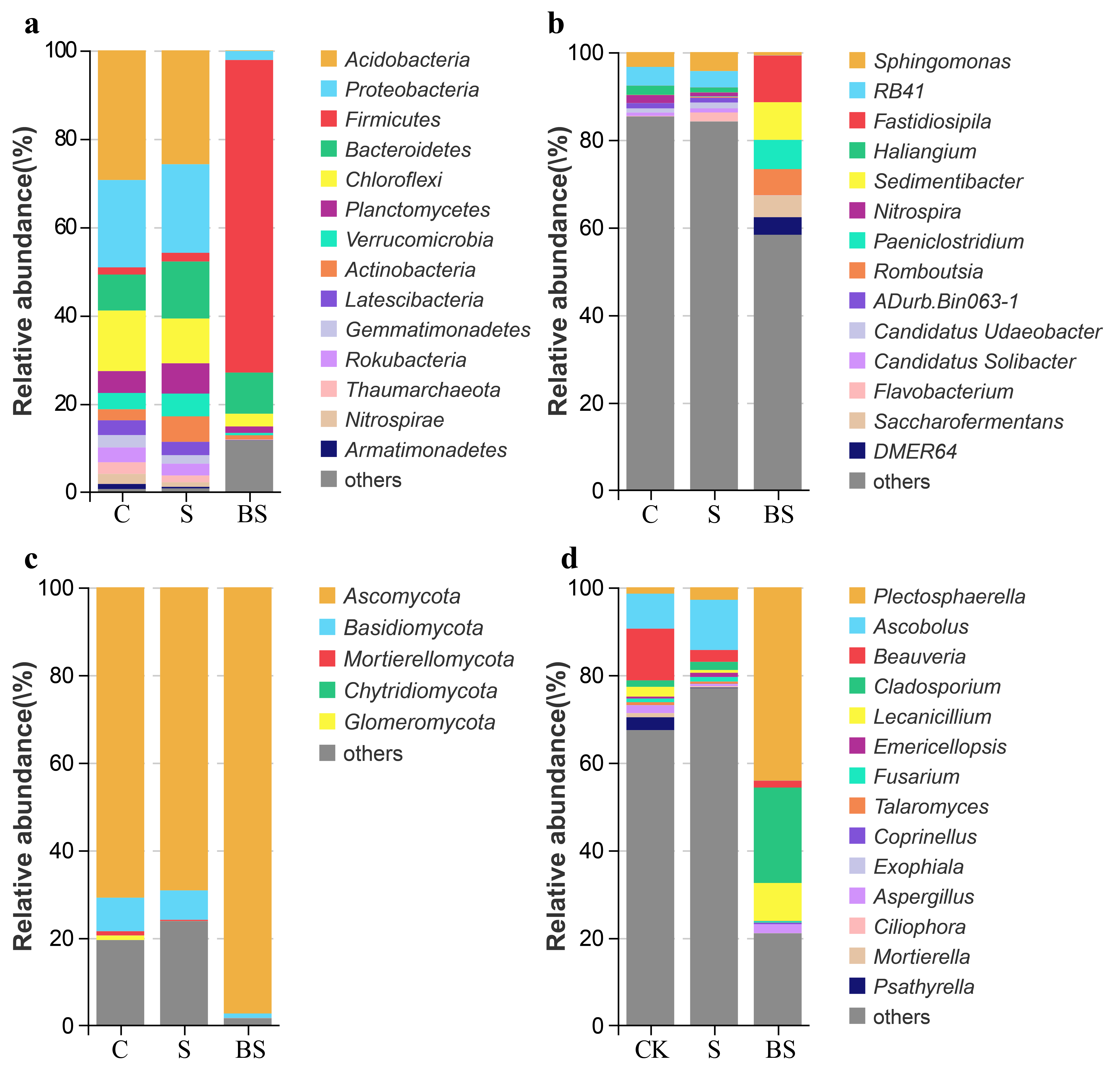

Supplement: Supplementary file 1 [file microorganisms-12-00716-s001.zip › manuscript-supplementary/Figure S3.jpg]
